# Human cloning as reproductive means in future: a qualitative thematic study of underpinning values

**DOI:** 10.3389/fpubh.2024.1243801

**Published:** 2024-02-13

**Authors:** Camille Castelyn, Werdie Van Staden, Michael S. Pepper

**Affiliations:** ^1^Centre for Ethics and Philosophy of Health Sciences, Faculty of Health Sciences, University of Pretoria, Pretoria, South Africa; ^2^Department of Immunology, Institute for Cellular and Molecular Medicine, University of Pretoria, Pretoria, South Africa; ^3^SAMRC Extramural Unit for Stem Cell Research and Therapy, Faculty of Health Sciences, University of Pretoria, Pretoria, South Africa

**Keywords:** human reproductive cloning, values, thematic content, assisted reproductive technologies, ethics

## Abstract

**Background and objective:**

The possibility of using human cloning to reproduce has been met with unease, shock, and prohibition in many countries, as well as the International Committee for Monitoring Assisted Reproductive Technology and the World Health Organization. Exploring the value judgments that underpin these and other responses to reproductive human cloning (RHC) was the objective of this study.

**Methods:**

In a qualitative design, this study explored values in their variety underpinning responses to RHC by conducting individual semi-structured in-depth interviews among nine scholars who were purposively sampled for contributing various perspectives. Thematic analysis was used to uncover qualitative contents systematically.

**Results:**

Regulation of RHC, the first theme, was valued highly but this should become more sophisticated than plain prohibition and draw on accountable societal engagement that is well-informed by current knowledge and further research, rather than be misled by for example the mistaken assumption that cloned offspring would be exact replicas. The second theme was about potential consequences of RHC for which engagement and regulations should account. It concerns the valuing of the personhood and dignity of offspring from RHC, and averting exploitation and potential unwanted societal consequences. In the third theme, participants valued the individual’s freedom to choose and reproduce.

**Conclusion:**

Recognizing the needs among people who cannot reproduce in other ways, the agenda for the societal engagement on RHC suggested by this study is extensive and challenging. It includes that potential consequences should be pre-empted, exploitation of RHC be averted, criteria of acceptability and non-acceptability of using RHC be developed, and the limits to the use of RHC be articulated in accordance with technological constraints and the values, resources and preparedness of societies.

## 1 Introduction

Reproduction has hitherto been dependent on the gametes of two people producing offspring either by coitus or through assisted reproductive technologies (ART) such as *in vitro* fertilization (IVF) and intracytoplasmic sperm injection. New technologies have opened various reproductive choices, such as using tissue banks to obtain gametes, *in vitro* gametogenesis, artificial wombs and three-parent babies (1–3), as well as the possibility of reproductive human cloning (RHC).

The possibility of RHC however has been met with unease, shock, prohibition, and exclusion among ARTs as currently defined by the International Committee for Monitoring Assisted Reproductive Technology and the World Health Organization (2, 4–6). Prohibition of human cloning features in several conventions, declarations, guidelines, policies, acts, laws and other legal instruments (4, 7–11). Intergovernmental organizations such as the United Nations issued a Declaration on Human Cloning, in which human cloning is prohibited, for “it...goes against human dignity” (4). Although the United Nations did not succeed in establishing a legally binding way to ban human cloning globally (7), many individual countries such as South Africa, Argentina and Canada have done so (12).

Prohibiting RHC is based on shared values regarding its safety and potential impact on society, the identity of individuals and views on different modes of reproduction (7). By these values, RHC should or must be banned. Values are also reflected in the responses of uneasiness, shock and appalment (13–16). A *Time* magazine article is concerned about the possibility of a human clone being a political candidate in future elections, “Could a clone ever run for president” (17). Another refers to the financial implications of human cloning, “Human Cloning: Recipe for next financial crisis?” (18) Values pertaining to the influence of reproductive cloning on an individual’s perceived identity also feature. A *Time* magazine cover questions pertinently, “Will there ever be another you: A special report on cloning.” (19). Another *Time* author writes about reproductive cloning changing people’s perceptions of their uniqueness, “Human cloning: copydog, copycat…I’ve never met a human worth cloning.” (20). In 2003, a group of doctors, scientists and legal experts, called upon the United Nations to seek a ruling from the International Court of Justice (World Court) to declare RHC, a “crime against humanity” on the basis that it would entail experimenting on human beings (21).

Values are evident in proclaiming that RHC is a “crime against humanity,” “narcissistic act” and that it would be exploitative similar to the experiments on humans during the Second World War, (21). Values also underpin comparisons between RHC and current reproduction choices (22, 23). A *Time* magazine publication questions whether therapeutic cloning will lead to reproductive cloning becoming accessible: “Cloning humans, the first laboratory duplication of a human embryo raises the question: Where do we draw the line?” (24). It has also been questioned whether reproductive liberty should be afforded equally to allow a prospective parent to choose to reproduce through reproductive cloning (23).

Legal values underpin reproductive laws as well as the rights and constitutional liberties that have bearing on RHC. The rights to freedom of expression and the doing of scientific research have for example been applied to RHC (25, 26). The right to reproduce and freedom to procreate have been interpreted in South Africa, for example, as extending to the use of ARTs (27, 28). This means that in so far as RHC is a form of ART, RHC may fall within the scope of this constitutional right.

Negative value judgments underpin the prohibition, uneasiness, shock and appalment regarding RHC (29). While apparent in the examples provided above, what the value judgments that underpin these responses to RHC are about, have not been examined systematically before. A qualitative enquiry is required to explore the variety of both positive and negative value judgments that underpin responses to RHC. The values by which for example RHC would be a “crime against humanity” and a “narcissistic act,” need to be explored and compared in terms of how these values would apply to using IVF, other new technologies, and coitus in human reproduction. To this end, this qualitative study systematically explored the values that underpin responses to RHC. Being a qualitative exploration, the themes that capture the variety of content were of interest, rather than the frequencies or general regularity of results for which a subsequent quantitative study may use the findings of this study in formulating hypotheses.

## 2 Materials and methods

### 2.1 Research design

A qualitative design was used to unpack the underpinning values in responses to RHC. It was premised on an interpretivist paradigm in which the subjective perspectives of participants were embraced and accounted for systematically (30). Critical theory was used to understand and co-construct the meaning that mattered to participants within their social structures and cultural practices (30).

### 2.2 Research participants and setting

Participants were purposively selected in seeking perspectives from a variety of professional backgrounds and interest in reproductive cloning. All nine participants had postgraduate qualifications in one or more of the following disciplines: medical science, molecular medicine, reproductive medicine, psychiatry, philosophy, health ethics, theology, journalism, law, genetics, and immunology. Three and six participants were, respectively, female and male. The study was situated in Pretoria, South Africa, and six participants were senior academic personnel affiliated at two large universities in Pretoria, aged 47 to 63, while the other three participants were in their thirties. Four of the participants have published scholarly articles in health ethics and teach this part time to medical and other students.

### 2.3 Data sources

Data were sourced through individual semi-structured in-depth interviews, supported by field notes and memos that we had made during the study. The audio-recorded, semi-structured interviews, lasting from 45 to 90 min, were flexibly guided by five to ten open-ended questions, thus aiming to uncover a variety of perspectives rather than to pursue consistency among participants. This was also the reason for conducting individual interviews rather than focus group discussions. Specific probes and examples were used to stimulate responses from participants, captured alongside the open-ended questions in an interview guide. For example, questions commenced with, “What has been your past exposure to the idea of human cloning” and “Considering your background and training, what are the concerns that you may have regarding human cloning?.” Thereafter more in-depth probes were used such as: “Why would human cloning for reproductive reasons be wrong/right/good/bad?” and “What would be valued by society and/or individuals in cloning humans for reproductive reasons?”

### 2.4 Data analysis

Following the verbatim transcription of the audio-recordings, data were analyzed by deploying the standard steps of thematic analysis (31). This involved several rounds of sorting textual contents through theoretical and axial coding until themes were derived iteratively that saturated all the data. Theoretical and *in vivo* coding were done by identifying the values that were expressed or implied in the text. Each sentential phrase and sentence that expressed a value word was coded for the specific value it expressed, capturing the intentionality or “aboutness” of each fragment. For example, value words such as “bad,” “poor,” “worse,” “worst,” “good,” “better,” “best,” “right,” and “wrong” were coded in association with the content as it was evaluated by the participant. Explicit and implicit expressions of values were differentiated in the coding. For example, an explicit “good” value was coded for “the good consequences of reproductive cloning would be personal fulfillment for people who could not have reproduced in a natural way,” whereas an implicit “good” value was coded for “reproductive cloning would open up infinite possibilities.”

Axial coding structured the data together in deriving higher level codes that connect codes progressively at higher levels of abstraction (32). The highest level of codes comprised the themes emerging from the analysis reflecting data saturation. The bracketing aspect of grounded theory was applied in the axial coding (33). This deliberately eschewed the potentially deleterious effects of the researchers’ preconceptions that could have tainted the analysis. Moreover, the criterion was applied pervasively in the analysis that higher level codes should express meaning as close as possible to the meaning of the raw content. Congruently, verbatim quotes that capture the themes most aptly are congruently provided in reporting the findings. The origin of each quote is marked by a unique alphabet letter for each participant and a code number assigned during the analysis to the fragment or sentence used by the particular participant.

### 2.5 Ethical considerations

This study was approved by the Research Ethics Committee of the Faculty of Health Sciences, University of Pretoria, study number 24/2019. All participants gave informed consent to participation in this research and each participant affirmed their informed consent on an ethically approved study-specific informed consent document. The study adhered to the 2013 version of the Declaration of Helsinki.

## 3 Results

Three main themes, their subthemes and a verbatim quote that captures each of these aptly are presented in Table 1. In the first theme, regulation of RHC was valued highly, but this should nonetheless allow for accountable engagement on the use of RHC in a society and in individual instances. Regulation should be more sophisticated than plain prohibition and engagement should be well-informed by existing knowledge and further research. The second theme was about potential consequences of RHC for which regulations and engagement should account. It concerns the valuing of the personhood and dignity of people reproduced by means of RHC, exploitation, and potential unwanted societal consequences. In the third theme, participants valued the individual’s freedom to choose the way in which they want to reproduce.

**Table 1:** Summary of findings regarding reproductive human cloning.

Themes	Subthemes	Salient examples
Theme 1: Both regulation and accountable engagement required	1.1. Regulation more sophisticated than plain prohibition through accountable engagement	“Simple prohibition is not indicative of mature society. I would hope for sophistication and maturity” (I5.3, I5.4).
	1.2. Well-informed engagement	“Educating people and addressing stigmas surrounding reproductive cloning and the myths surrounding people if people do decide to do something that they are clear on what it actually is and what the primary purpose then would be” (H7.2).
Theme 2: Potential consequences for which regulations and engagement should account	2.1. Valuing personhood and dignity of a clone	“A person has value in itself not because of their genes or how they were created” (H5.4). And “...Even if I clone myself the person is not going to be the same as myself, because they are going to have different experiences in life and they will not be the same person. Yes but I think to artificially clone somebody is different.” (F14.1).
	2.2. Exploitation for unacceptable selfish reasons and corruption by people in powerful positions	“It would be important to regulate because individuals are easily corrupted by their own (selfish) desires.” (H6.2).
	2.3. Unwanted societal consequences	“The decision to then clone themselves would, in all likelihood, be because of their narcissism, and then you would just – there will be a greater percentage of people with that problem thinking about themselves and not for the greater good in society. So, I think, yes, in the end, you would sit with individuals with certain characteristics and there would not be a diversity of characteristics, and I do not think that’s good, because a lot of the characteristics that you would then sit with are not necessarily positive” (F19.2).
Theme 3: Freedom and wonder to reproduce	3.1. Reproductive freedom to become a parent by whichever means	“Based on individual freedom and a libertarian society there should not be any limits to reproductive freedom, You know I find it difficult, because I do not think that we want to live in a prescriptive society. I do value individual freedom. I think that is incredibly important, it’s one of the core values. The freedom to explore what is valuable for you and without too many restrictions” (E18.1).
	3.2. Reproductive freedom with limits	“I think your freedom only goes that far, but I think there are limits.” (E18.2).
	3.3. Reproductive freedom has to do with the wonder of reproduction	“The ethical debate around cloning, *per se*, does not concern me as much – humans are, by nature, an inquisitive species” (B3).

### 3.1 Both regulation and accountable engagement required

While all participants underscored the crucial need for regulating RHC, regulating RHC may be developed in ways that provide for the value complexities of RHC better than a plain prohibition. To this end, accountable engagement at societal (or general) and individual (or *ad hoc*) levels was suggested. This engagement regarding RHC should entail educating people and quashing myths and mistaken expectations of RHC.

#### 3.1.1 Regulation that is more sophisticated than plain prohibition through accountable engagement

Participants reflected on prohibition as currently the dominant way in which RHC is regulated, citing Chapter 8 of the South African National Health Act 61 of 2003 (A31) and stating “Prohibition of reproductive cloning is the dominant view currently” (I2.2). Participants considered prohibition as a justified regulation at this time, mainly owing to safety concerns (C14.2, E17.2, H10.5) and because “It’s far too difficult and complex” (A30). However, participants also deemed it sensible to reconsider this even though they were not prepared for prohibition to be repealed at this time, particularly in the absence of more developed regulation that may substitute prohibition. While some participants were unable to choose between prohibition and acceptance of RHC in current times (D11.1, D12.2), regulation varying in extent was considered suitable. That prohibition was the dominant way of regulating RHC thus obscuring more nuanced regulation was expressed in the concern that the definitions of reproductive cloning in South African National Health Act were “archaic” (A33, I2.2). Another concern was whether reproduction could truly be prohibited without infringing on a human right to freedom: “We have the right to choose, and this would be important even though no matter what we did in the past, we should never be subjected to not have a choice” (H20.5). Instead of plain prohibition, the view was expressed that “simple prohibition is not indicative of mature society. I would hope for more sophistication and maturity [than that]” (I5.3, I5.4). Finding middle ground despite complexities was mooted: “You know, how do you regulate in an area which is so complex such as this? Some countries have different regulatory approaches. Some countries take a strict control approach; they assume it’s dangerous… Others are the other way around, so we have a middle ground” (A32).

Finding a middle ground applies to the formulation of regulations that are “more sophisticated” than plain prohibition by means of accountable engagement among the stakeholders of a “mature society” (I5.3, I5.4). It also applies to the application of more sophisticated regulation in individual instances, on “a case-by-case basis,” when reproductive cloning would be acceptable and the reasons why it would not be (D18.3, G11.3). For example, “if parents wanted to decide to reproductively clone themselves as a means of reproduction, there would have to be certain guidelines, not as stringent, but within certain guidelines of what is acceptable and what is not acceptable” (H6.3). Accountable engagement and sophisticated regulation for both policy purposes and the execution at the individual level would thus enable RHC beyond the absolute exclusion of any meritorious situation imposed by prohibition.

#### 3.1.2 Well-informed engagement

Accountable engagement toward more nuanced regulation and individual decision-making regarding RHC should be well informed. To this end, the appeal was “We must first have more knowledge” (D12.2, C12.2). Owing to our limited knowledge especially regarding potential consequences, it was recommended that we should be “erring on the side of caution” (C13.7).

Engagement should furthermore entail “educating people and addressing stigmas surrounding reproductive cloning and the myths surrounding it...for people to decide to do something that they are clear on what it actually is and what the primary purpose then would be” (H7.2). Educated engagement will help in dealing with the problems of misinformation: “The way in which we need to evolve and move forward and deal with the problems that we deal with is through education. So, …one of the responsibilities that we have collectively is we need to inform people, because part of the opportunity of accessing these things comes through understanding them, or at least being aware of them” (C21). When people are better informed, they would be in a better position to evaluate the various perspectives on RHC: “People need to have access to information that will at least allow them to be aware of what is available and I mean I am not even talking about them being aware of the positive effects or the side effects, the positive and the negative, but just to understand that there is something there that is being done that may benefit them or their families or their children at some point in the future” (C21).

### 3.2 Potential consequences for which regulations and engagement should account

In the first theme, sophisticated regulations and accountable engagement were valued highly. That for which regulations and engagement should account, surfaced in the second theme. It concerns the potential consequences of RHC in the valuing of personal identity and personhood of people reproduced by means of RHC, potential exploitation, and unwanted societal consequences. According to this theme, exploitation, corruption, and RHC for unacceptable selfish reasons should be averted and potential unwanted consequences for society should be anticipated and accounted for.

#### 3.2.1 Valuing personhood and dignity of cloned offspring

As an important consequence of RHC, participants raised the issue of personhood and identity: “it is very interesting, the sense of identity feature again with … reproductive cloning” (H15.1). Participants anticipated that it would be “a big question” whether societies would ascribe personhood, value and dignity to “a clone” (E13.2, H5.1, H6.1). Participants maintained however that the way in which someone was reproduced should not determine whether they qualify as a person with value and dignity: “the means by which we reproduce does not matter” (H9.1) and “a person has value in itself, not because of their genes or how they were created” (H5.4). Participants justified this stance by comparing RHC, IVF and coital conception: “Comparing IVF with reproductive cloning one could say that in both cases the child would be more wanted and consciously decided for than with normal reproduction due to money and time being spent” (H5.1).

Participants alluded to misperception, mistaken portrayal and stigmatization that may influence societal acceptance of the personal identity and personhood of people reproduced by RHC. The issue of a clone being an exact replica as in the following statement to “reproduce an exact clone of themselves” (H5.2), was provided as a good example. Whether “the clone” is understood to be an exact replica would influence the ascription of personal identity and personhood. Participants maintained however that a clone “would not have the same identity as the original, due to environmental and parental upbringing influences” (D11.1, G15.1), and “I personally do not think, for example, that if I were to be cloned, that I would be 100% the same person. Because my environment, my upbringing and all of that other metadata factors, so there is, I think, definitely, there will be differences, but that it would be the same me, I doubt it” (G14.4). Another participant affirmed this as a misunderstanding of the biology and technology: “There is a misperception that because someone has identical genetic makeup, they are identical people” (I3.5). There was furthermore a comparison drawn between parents’ own identity and the unique identity of their own children: “No more than our children would be an extension of ourselves [would a clone be an exact replica]” (I8.2).

Participants also alluded to RHC influencing whether a child would be “wanted.” Children produced by cloning may be more “wanted” than naturally reproduced children. They gave the example of parents deciding to have a child using IVF and that similarly, a child born through RHC may be “more wanted” than naturally produced children, because prospective IVF and RHC parents would have to invest more time, money and effort into producing a child: “Comparing IVF with reproductive cloning one could say that in both cases the child would be more wanted and consciously decided for than with normal reproduction due to money and time being spent” (H5.1).

Being “wanted” may also influence the cloned person’s identity and perceived personhood. A participant said that every child, no matter how they are produced, needs to feel valued. The feeling of being valued links to a feeling that the people who brought them into this world wanted them (E20.2). Another participant referred to the cloned person’s identity as a sense of individuality: “Also individuality, so every person must feel like they are a unique individual in their own right” (F18.3). This was contrasted with the many “unwanted children” born daily at a local hospital: “We have done studies … where more than half of the babies were unplanned and a lot of them were unwanted, that the women actually did not want to have” (E20.2).

#### 3.2.2 Exploitation for unacceptable selfish reasons and corruption by people in powerful positions

It was suggested that sophisticated regulation and accountable engagement could address concerns that reproductive cloning might be exploited for unacceptable selfish reasons and that it may be corrupted by people in powerful positions if it were not well regulated. This was aptly expressed as “it definitely needs to be managed, it should not be like I decide I want to clone myself and then I do it. There should be a greater control process on why, for what – questions that I think are important to the discussion” (C11.3). Reproductive cloning can be exploited for unacceptable selfish reasons when the decision to clone is based on unacceptable self-interest and even a narcissistic pursuit, captured for example as “It would be important to regulate because individuals are easily corrupted by their own (selfish) desires” (H6.2) and “I am not sure whether, morally, it’s the correct thing to do, because I think people will do things for selfish reasons” (F14.2). Selfish reasons included the quest for immortality (F14.3): “selfish reasons – a lot of people will say, ‘well, I do not want to really die now, so I will clone myself and see,’ you know, because there is the unknown about that. Maybe if I reproduce myself, I will continue in existence – things like that” (F14.3). Another reason that was described as selfish (H6.2) was the quest for beauty: “Reproductively cloning for the pursuit of beauty is a very selfish outlook on what the meaning of life is or what is valuable in our society” (H11.1). Participants compared RHC to coital reproduction, some remarking that both might be selfish. Examples were that both means of reproduction could be used to produce a “mini me” (H3.3), “increase your value as a woman” (E16.1), or in “the yearning to leave something behind” (G14.4). Notwithstanding these similarities, participants valued coital reproduction in which two people are involved as being less selfish than cloning a single person: “Natural reproduction is something beautiful and the ability just to make more of oneself [in RHC], [in doing] that we become a bit more selfish and self-driven” (G12.1). There were concerns that RHC could be exploited by people as a function of their narcissistic or psychopathic personalities. Some participants considered the possibility that a psychopath may choose to clone themselves: “It is not conducive if a psychopath clones himself, so as to terrorize everyone.” Participants nonetheless reflected on the complexities of this: “There is a fine line, because I may think this person is a psychopath and another may disagree, so again the question of who determines the criteria etc. … reproductive cloning should be a regulated thing” (G11.2). Moreover, another participant observed that narcissistic people are not necessarily bad people, they may even have done well for themselves: “Well it’s not necessarily all bad, I think there are lot of narcissists because they are narcissists they have done well and they have advanced human life in a certain way” (F20.1).To prevent exploitation of RHC as a function of psychological problems, it was suggested that people should be screened psychologically before being allowed to reproduce by RHC: “I would say that people should all first get psychological assessment or psychiatric assessment before they are allowed to be reproductively cloned, but I actually also feel that people who naturally reproduce should also undergo assessment” (H10.5).Concerns with unwanted psychological influences pointed to the more general questions: “Who is worthy or who is not worthy” of being cloned (H10.3, H10.4), and who decides this? People in powerful positions may decide this, which would be “dangerous” owing to potential exploitation and corruption: “Who decides who can reproductively clone themselves? That is dangerous who decides who is worthy and who is not worthy.” Using RHC for producing extra organs was an example of exploiting RHC that participants considered: “People who are narcistic in the end tend to have a lot of difficulties in life that they struggle with, because of them, their inherent default functioning of thinking about and doing everything for themselves. And the decision to then clone themselves would in all likelihood be because of their narcissism and then you would just – there will be a greater percentage of people with that problem thinking about themselves and not for the greater good in society.” (F19.2). Their “inherent functioning” to survive could be endangered so that they are coerced to clone themselves for purposes such as providing organs for others (F19.2). This could be dangerous for people who already have “a lot of difficulties in life that they are struggling with” (F19.2). It was remarked that clones with the purpose of organ donation would be extremely beneficial from a medical-scientific perspective because the clone would be a perfect donor match, eliminating many side effects and risk factors involved with organ transplantation: “In terms of organ donation and that type of thing, I think there are some questions…then we would have new hearts etc.…” (G12.1). This “transaction” would be beneficial to the person who was cloned, but would be exploiting the person who resulted from the cloning: “Just to make more of oneself [in RHC], [in doing] that we become a bit more selfish and self-driven” (G12.1). and “A piece of narcissism: yes and I want to be the one who is cloned [and not someone else]” (G12.3).

#### 3.2.3 Unwanted societal consequences

RHC may have consequences not only for individuals but also for the larger society; “small decisions may lead to bigger issues” (D17.4) The potential consequences add complexity to the sophisticated regulation of, and accountable engagement on RHC: “That I find even more complex” (E13.2). Consequences of RHC for society may “not [be] for the greater good” (F19.2) and may be bigger than humanity is able to comprehend at the time (D17.2).

RHC could fall into the wrong hands, disturbing the balance in society: “I think, if it becomes out of balance” (F20.1) and “There are a lot of these people around, and what you do not want to do is put the tool in their hand to allow them to do this, so that they end up creating a race of people” (F20.1) Similarly, another participant mentioned the potential creation of a clonal population who would not necessarily have “positive characteristics” (F19.2), as when for example RHC is used predominantly by narcissists who want to clone themselves: “The decision to then clone themselves would, in all likelihood, be because of their narcissism, and then you would just – there will be a greater percentage of people with that problem thinking about themselves and not for the greater good in society. So, I think, yes, in the end, you would sit with individuals with certain characteristics and there would not be a diversity of characteristics, and I do not think that’s good, because a lot of the characteristics that you would then sit with are not necessarily positive” (F19.2). Potentially disturbing societal balances was also captured in: “What I do not want to do is create a clonal situation where you have got people with a particular trait which you think is better, but you cannot measure the long-term consequences of that against everything else that’s being ignored. I think all these things need to go forward in a positive way, and we cannot really do that at the moment” (C8.2b).

Another unwanted consequence that was raised was that in the pursuit of beauty, RHC could shift perceptions of that which is considered beautiful: “it is a very bad idea to reproductively clone in the pursuit of beauty. We should not encourage a couple where the one is really beautiful, the other not so beautiful, to not reproduce naturally but rather reproductively clone so as for the beauty to prevail the appearance of future generations… Reproductively cloning for the pursuit of beauty is a very selfish outlook on what the meaning of life is or what is valuable in our society” (H10.6, H11.1).

### 3.3 Freedom and wonder to reproduce

The third theme concerns the freedom to reproduce, which was expressed as a right to become a parent by whichever means one may choose, yet also as a freedom that is limited. Participants connected to this freedom, the wonder (or amazement) of reproduction.

#### 3.3.1 Reproductive freedom to become a parent by whichever means

For some participants, reproductive freedom included the right to become a parent, by whichever means they choose. The means could include ART such as IVF and even anticipated RHC. A participant said that there should be no limits to reproductive freedom: “Based on individual freedom and a libertarian society, there should not be any limits to reproductive freedom. You know, I find it difficult, because I do not think that we want to live in a prescriptive society. I do value individual freedom. I think that is incredibly important; it’s one of the core values – the freedom to explore what is valuable for you and without too many restrictions” (E18.1). Participants related the freedom to reproduce by cloning to that of IVF: “I think one should make comparisons between reproductive cloning and IVF” (I5.1, I5.5). Similar to IVF, this freedom was expressed as “I could identify with the situation if a person was single, giving up on the quest for a partner and resort to cloning themselves” (H4).

The freedom to utilize RHC was also considered in terms of rights – both the right to reproduce and the limitations to the right to have access to it as a healthcare service. This was expressed as “…it’s a second generation fundamental right, and the way it’s interpreted is normally the state only needs to implement or realize that right as it is reasonably able to do so. So, if the state does not have the resources, it will only need to accommodate implementing the rights within reasonable limits, because it’s all dependent on the state budget, ultimately. So, it’s there, but it has built-in conditions.” So qualified, access for all was doubted: “The basis for a decision of this nature should always be one of access. Universal access. And that would never be the case” (B22).

#### 3.3.2 Reproductive freedom with limits

Some participants attached limits to reproductive freedom: “I think your freedom only goes that far, but I think there are limits” (E18.2). Comparisons were made with the restriction imposed by China’s one child policy from the late 1970s until 2016 (F10.1). A limit was that other means of reproduction should take precedence and that RHC was only acceptable as a last resort for couples who had no other means by which to reproduce (H3.2; F15.2). RHC should furthermore not be harming people: “I think, obviously, as I said, I try and stick to something like if it’s going to harm other people when we as society we can say, look, this is not acceptable. But if there’s no clear, direct harm and it’s mostly self-regarding, then I think people should have the freedom to do a lot of things” (E18.2). Other limits were that it should not be bad for the environment (E19.3), nor should it pool large amounts of resources for personal satisfaction (E19.3).

#### 3.3.3 Reproductive freedom has to do with the wonder of reproduction

Reproductive freedom was connected to the wonder (or amazement) of reproduction that may be forfeited using RHC on the one hand, but may also be inquisitively explored and pursued through RHC. Reproduction was described as the “miracle of natural life” (G5.2, G12.1), that “There is something inherently beautiful about two people getting together to reproduce, it also shares decision-making responsibilities right from the beginning” (G12.2) and “I think it is something that needs to be treasured at all costs, something holy, the miracle of life, that we actually do not just make more of ourselves, then we might become self-driven and selfish” (G12.1).

Owing to the wonder of reproduction, restricting freedom to use RHC was considered as potentially futile because humans are inquisitive by nature and will explore this anyway (B3, I2.3). This was for example expressed as “The ethical debate around cloning, *per se*, does not concern me as much – humans are, by nature, an inquisitive species” (B3).

The wonder of reproduction through RHC was about new possibilities: “I am not as skeptical about reproductive cloning; I am rather positive about the opening up of possibilities” (I2.3) and “the good consequences [of RHC] would be personal fulfillment for people who could not have reproduced in a natural way. That could be a good outcome” (H9.5). The possibility of reproducing using RHC for single people, infertile couples, or people with genetic conditions (H9.5), who have no other way of reproducing would improve their quality of life and sense of purpose (H4, H9.5), stated also as “Well, is not that something that we – well humankind has advanced always, throughout the years, and I think human advancement in general improves quality of life” (F16.4).

## 4 Discussion

Participants in our study valued both the regulation of RHC and the freedom to use it but the shared values by which to regulate RHC, particularly if prohibited, conflict with the value ascribed to the freedom to choose and reproduce by means of RHC. This tension may be addressed, our findings suggest, through accountable societal engagement from which regulation should be developed that is more sophisticated than plain prohibition.

**Figure 1:**
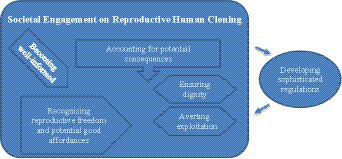
Societal engagement regarding reproductive human cloning suggested by the themes.

Captured in Figure 1, the societal engagement suggested by the findings will require a process in which society and the stakeholders become well-informed, the good affordances of RHC are recognized, the consequences are pre-empted and accounted for in the decision-making process, specifically in ensuring that the offspring reproduced by RHC are bestowed with personhood and dignity, and that exploitation of RHC be averted. Societal engagement on RHC may be guided by well-developed and seasoned practices in health policy development (34) and attaining accountability standards in extensive stakeholder engagements (35).

It will rather be early days for this societal engagement process, being at first explorative regarding the various issues raised in our findings, no less so owing to the controversy, sensitivity and conflicting values that people hold dearly regarding RHC. Conscious of this values diversity, this process should account for differences between values as described elsewhere (29, 36, 37). The process at first will need to decipher the shared values from values that are legitimately diverging and even conflicting, with the view to capture the shared values in mooting a policy framework and formalizing regulations, including transitory regulations. These sophisticated regulations will accordingly express the shared values as a framework within which to provide for the diverging values regarding RHC and regulated processes for further societal engagement.

Crucial for the success of a societal engagement process will be that all stakeholders become well-informed about RHC. Our findings suggest that RHC is commonly misunderstood and subject to myths, in particular that cloned offspring would be exact replicas and not be human persons with subjective experiences including an own identity. Assuming that the cloned offspring would be an exact replica often underpins the evaluation that RHC would be wrong (38). Accurate information on RHC will be important no less so than is already established in guiding society and prospective parents on ART (39). Misinformation may result in misconceptions, poorly made choices and even induce stigmatization (2).

Through societal engagement several ways should be developed to ensure that the offspring reproduced by RHC are bestowed with personhood and dignity the same as everyone else. One way is to expose as false the assumption that these offspring would be replicas. Bestowing personhood and dignity for cloned offspring is congruent with the established norms for IVF and other ART. The personhood and dignity of someone reproduced using IVF, other ART or adoption, are not in question, and whether ‘natural born’ has become a non-issue in the 40 years since Louise Brown was the first born through IVF (40). Similar comparisons may be made regarding the issues of disclosing one’s reproductive “identity” as originating from for example RHC, IVF or adoption. Disclosure of cloned status will need to be considered, as an individual’s experience of identity may be influenced and conversely, the societal perceptions may influence the identity of cloned offspring similar to the way in which societal perceptions have influenced perceived identity of children born through IVF, intracytoplasmic sperm injection, or have been adopted (41, 42). In any event, ethical deliberations about the issues of disclosure, and RHC more generally, should fundamentally be steered by the human dignity of people regardless of how they have been reproduced in line with the United Nations’ Universal Declaration of Human Rights (43).

The agenda for societal engagement on RHC is extensive and challenging. Our findings suggest that potential consequences should be pre-empted through societal engagement. This engagement should lead to processes that account for the various potential consequences in policies and regulations according to the values attributed to the consequences. These processes and eventual regulations should avert exploitation of RHC, should develop criteria of acceptability and non-acceptability of using RHC, and articulate the limits to the use of RHC in accordance with technological constraints and particular society’s values. Processes and regulations should also plan for the monitoring of and responses to unforeseen unwanted consequences. Included in this agenda, our findings suggest furthermore that RHC shares the challenges of inequity that healthcare services currently experience at large (44). Policies to address these may be developed along established decision-making processes and particularly through societal engagement (34).

Embarking on societal engagement regarding RHC is impelled by virtue of recognizing the freedom to reproduce and that RHC is opening up new possibilities particularly for the quality and purpose of life among those people for whom other means of reproduction may not be available or health impairments pertain (e.g., inheritable genetic disorders). In a person-centered ethos (45, 46), the needs of these people for RHC are paramount rather than scientific or commercial interests that may drive new RHC technologies.

### 4.1 Limitations of the study

Uptake and feasibility of the societal engagement suggested by our study will vary depending on regional and global governance constraints on RHC (7, 8, 10). Societal engagement and RHC itself, are furthermore dependent on various resources including the maturity of societies to embark on these processes and develop policies and regulations accordingly. One may reasonably expect that extensive societal engagement and RHC will be out of reach and even too daunting in many contexts where plain prohibition of RHC may indeed be more suitable at this time. Nonetheless, our findings foreground the need for RHC among people who cannot reproduce in other ways, and suggest that people should be better informed, dispelling the myths regarding RHC portrayed in the media (13).

Being a qualitative study, the findings of this study were meant to capture a variety of content, that is, what the values pertaining to RHC were about in their variety, rather than the frequencies or general regularity of the results as would be applicable for a quantitative study. Investigating how commonly the themes and subthemes feature in various populations would require subsequent quantitative studies. However, even in the absence of such quantitative results, a case may nonetheless be made for the findings of our study as relevant and important in a specific context based on societal interests or ethical grounds. For example, societal and ethical grounds seem obvious enough in making a case for well-informed societal engagement and dispelling misleading perceptions regarding RHC.

The variety of content yielded by the study was constrained by the saturation of themes in the qualitative analyses, which means an exhaustive account of what values on RHC are possibly about, could not be attained considering the nature of this topic involving in part speculation about potential consequences. Subsequent qualitative studies may accordingly be expected to reveal other issues regarding values pertaining to RHC.

## 5 Conclusion

This study recognizes the tension between current prohibition of RHC and the new possibilities that it holds for the quality and purpose of life of those people for whom other means of reproduction may not be available or who suffer from an inheritable genetic disorder. Amidst this tension, our study suggests that the way forward is through accountable societal engagement on the topic through which policy and regulations may be formulated.

Impelled by reproductive freedom and equity, our study suggests that the agenda for societal engagement on RHC is extensive and challenging. It includes themes highlighting that potential consequences should be pre-empted, exploitation of RHC be averted, criteria of acceptability and non-acceptability of using RHC be developed, and the limits to the use of RHC be articulated in accordance with technological constraints and the values, resources, and preparedness of societies. Crucial for the success of societal engagement is that all stakeholders should be well-informed on RHC, drawing on existing knowledge and further research, instead of mistakenly assuming that cloned offspring would be exact replicas. Thus informed, societal engagement should ensure that personhood and dignity are bestowed on cloned offspring in the same way that personhood and dignity are not in question for children who have been reproduced using IVF, other ART, or who have been adopted.

## Data availability statement

The raw data supporting the conclusions of this article will be made available by the authors, without undue reservation.

## Ethics statement

The studies involving humans were approved by Research Ethics Committee of the Faculty of Health Sciences, University of Pretoria, South Africa, study number 24/2019. The studies were conducted in accordance with the local legislation and institutional requirements. The participants provided their written informed consent to participate in this study.

## Author contributions

All three authors were involved in the conceptualization and the execution of the study. CC conducted the interviews and drafted the first manuscript. The analyses were executed by CC and WS. All three authors developed the manuscript iteratively and endorsed the final version.
